# Reminiscences of the Effects of Chlorine, Largely Inhaled, with the Favourable Result of Copious Bleeding

**Published:** 1848-04

**Authors:** John Redman Coxe


### MEDICAL EXAMINER,

AND

### RECORD OF MEDICAL SCIENCE.

NEW SERIES.—No. XL.—APRIL, 1848.

### ORIGINAL COMMUNICATIONS.

### *Reminiscences of the effects of Chlorine, largely inhaled, with the favourable result of copious bleeding.* By JOHN REDMAN COXE, M. D.

In looking over some old papers long since laid aside and forgotten, I lately discovered the minutes of a case of considerable interest to me, inasmuch as I was, myself, the subject of which it treats. I had formerly intended to draw it up in detail, as not undeserving of notice by the medical profession; but having so carefully, although unintentionally, placed those minutes away, I have hitherto neglected it,—not choosing to depend on my memory of the occurrence, after a lapse of nearly 35 years,—and it is only within a few weeks that I have accidentally recovered them.

It is well known that Chlorine (at the period adverted to, known by the term of dephlogisticated, or oxygenated muriatic acid gas) is a gaseous fluid, absorbable by water, and possessed of a powerfully pungent and suffocating odour; so very remarkable, as scarcely to be mistaken for any other substance. It is totally unfit for respiration, and has been productive of death by its incautious inhalation. The celebrated Pelletier had the hardihood (if I may so speak of that illustrious chemist) to respire it, but fell a victim to his temerity, for it induced a rapid consumption. In even a moderate dilution of it in the atmosphere, it induces cough and catarrh, and animals plunged into it in its pure state are destroyed by it, and die, even before asphyxia is produced by the black and un-arterialized blood.*

In my course of Chemical Lectures in the University of Pennsylvania, during the session of 1811—'12, I had, in the order of the course, prepared a considerable quantity of this gas, in illustration of my lecture of the succeeding day, and the process was progressing when the class assembled. Several jars filled with it were on the shelf of my water bath, and the retort employed, of about two quarts measure, was yielding it to other receivers successively, during the delivery of my lecture. At its conclusion, as usual, many of the students flocked around me to observe the process, the larger portion proceeding to the room of the succeeding Professor. Engaged in replying to their numerous questions, whilst holding the retort and directing the escape of the gas with which it was filled, my attention was drawn from its immediate object, and by some unfortunate movement, the retort was jammed against the bath and shattered to pieces, the gas immediately escaping and completely enveloping me. In endeavouring to avoid this noxious material, I augmented the mishap by knocking over and breaking the receivers, and again receiving their full contents over my whole person. The gas speedily diffused itself around in every direction. As quickly as possible, the students retreated, and I was left entirely alone, with the unpleasant reflection that my preparations for the following day were entirely destroyed. Although my respiration was almost instantly affected on inhaling the gas, and a stricture of the fauces was quite perceptible, I hoped it would be soon so diluted by the atmosphere as to enable me to resume my process. I had, however, proceeded but a few minutes, when I was forced to cease, finding my lungs charged with this noxious gas, and that the difficulty of breathing was increasing, with an apparent constriction of the glottis, and an uneasy and dizzy sensation of the brain. I was led, from these symptoms, to feel my pulse, and found it weak and tremulous, leading me at once to cease all further attempts, and to proceed home as speedily as possible, being satisfied that I should soon sink under the influence of the gas, both as existing throughout

* See Dict. des Sci. Med. Article Chlorine; and Encyclop. Americana, &c.

the atmosphere and from that with which my lungs were replete. It was with difficulty I reached home, being in no wise relieved by the fresh and pure air; and I might, from the unsteadiness of my walk, have well been deemed to have taken an excess of spirituous liquor. I became now much alarmed, since, with an increase of the symptoms above mentioned, I found my pulse nearly reduced to a thread and diminished in its pulsations. All this, however, did not prevent me from speculating on my situation, and proposing to myself the question as to what measures I should recur for relief. I had no similar case in my recollection, nor of any mode of treatment that had been recorded; all I knew was that death had resulted from the respiring chlorine, either immediate or remotely, and I judged it might be my fate to add to the list. Whatever was to be done required instant execution. Blisters and evacuants, &c., I regarded as of a nature too slow to be beneficial; and considering that my lungs were replete with a fluid incompatible with beneficial respiration, and therefore that it was obvious the circulation of the blood through the lungs must be greatly impeded, as evinced by the pulse, and being thereby necessarily precluded from arterialization, I came to the conclusion that venesection could alone answer the end in view, and that, carried out to the fullest extent, as the only means by which complete engorgement of the lungs could be prevented, and myself possibly saved from speedy death from asphyxia, or from a species of peripneumonia notha. Under these impressions I reached home. My appearance alarmed my family, and anxious inquiries were made as to what was the matter. As no time was to be lost, I desired no questions to be asked, but to send for the nearest bleeder as quickly as possible, and that I would subsequently afford an explanation. The bleeder arrived in a few minutes; my pulse being now almost imperceptible, and my pulmonic oppression increased, with stricture and harsh dry cough. He tied up my arm, and I directed him to bleed me until I should order him to cease. My veins are very large, so that when I had occasion to employ venesection, I usually bled myself; but now, the veins were with difficulty distended, and the blood issuing from the orifice, slowly oozed out and trickled down my arm, of (as I expected) a dark, nearly blackish hue, resembling molasses in consistence. I was nevertheless thereby encouraged in the opinion

I had formed, but I found no relief to my feelings, or change in the fluid, when nearly twenty ounces were discharged. By this time the bleeder was beginning to be alarmed, and wished to tie up my arm, but I ordered him peremptorily to proceed. In this I was somewhat encouraged by a slight supervening nausea, which inclined me to hope that some change was about to ensue; it however soon subsided, and when upwards of thirty ounces had been drawn off, I began to experience a small relief from the oppression of my lungs and head, although my pulse continued its thread-like character, and the blood, in its flow and appearance, continued as before; nor was it until nearly forty ounces were obtained, that I could perceive any change in the pulse as to frequency or strength, nor in the sluggish flow from the orifice of the vein; a few more ounces discharged, had, however, the effect of unloading the vessels, and the pulse was obviously rising, yet with no alteration in the colour of the fluid; but I was now confident that in a short time my lungs would be relieved, and a free circulation thereby restored. Such was the fact, but more than forty ounces were in the basin ere I was gratified in perceiving a slightly vermillion tint usurping the molasses hue of the blood. This rapidly increased, and in a few seconds the hitherto stagnant fluid burst forth in a full and rapid stream, the veins became turgid, the relief of my lungs was greatly improved, and my respiration nearly free. Fearing that all the former symptoms might resume their sway, I determined still further to evacuate the vessels, more especially since I perceived my dry cough was giving way to a free discharge from the lungs, and I would not arrest the bleeding until nearly or quite sixty ounces had been drawn off. Greatly to the satisfaction of the operator was my order to stop the flow and bind up the arm; this he had no sooner effected, than he speedily decamped from whence he had been so thoroughly frightened I experienced but little sense of weakness from this free evacuation, but I was prevented from rest, on retiring to bed, by the continual cough and copious discharge of phlegm. This cough, in the course of an hour or two, set my arm bleeding again, whereby I lost probably five or six ounces more of blood, and with much relief. I confined myself to simple drink, as barley water, &c., and at night, by the aid of some sweet spirits of nitre and paregoric, I rested better than I had anticipated. The morning found me greatly relieved in all respects; though the cough and expectoration were much diminished, they did not altogether cease for nearly a week,—and after a slight breakfast, I felt so much improved, that, in opposition to my family, (and, perhaps, to prudence,) having no apprehension of any further danger, I determined to go to the University. I did so, and delivered my lecture, which was only rendered difficult or interrupted by my cough. When this entirely ceased, I found my lungs altogether free from the slightest affection; and now, after nearly forty years, they are vigorous and healthy, as in the period of my youth, having never during that lapse of time suffered the slightest pulmonary affection as the result of my unfortunate mishap.

Having been thus particular in detailing the circumstances of my own case, as a caution to others, and to point to the necessity of prudence in experimenting with this extraordinary agent, and with its congeners—such as "Chloroform"—(a barbaric term,) I may be allowed to ask, what would have been the result of any other mode of treatment? What other, indeed, could possibly have had a favourable termination? If speedy death had not followed the state of asphyxia into which I was rapidly hastening, any other plan, I doubt not, could only have prolonged life for a short interval; whilst disorganized lungs, leading probably to tubercular consumption, would inevitably, in a few weeks, have carried me to the grave. Reviewing my case, then, both physiologically and pathologically, my conviction is complete, that nothing but the prompt and copious discharge of blood could possibly have saved me.

Whilst on this subject of chlorine, I will embrace the opportunity of stating another instance of its effects, occurring likewise in my own person—not in its gaseous state, but in that of a concentrated aqueous solution, and at a subsequent period, evincing its prodigiously powerful and rapid action on the Schneiderian membrane. During one of the weekly examinations of my class, a Woolfs' apparatus, in which I had been preparing the gas, was left to itself; the extrication of the gas ceasing by removing the lamp from under the retort, the gas was absorbed by the water in the range of receivers connected, and allowed the water from the furthest to pass back into those nearest the retort, in consequence of the vacuum produced. Desirous of expediting the renewal of the process, I replaced the lamp, and, without reflection, very foolishly undertook to draw off some of the air in the remote receiver by applying my mouth to the tube of safety, thereby to diminish the pressure, so that the foremost receivers might cause the fluid that had passed into them more readily to regurgitate: the bent tube serving as a valve by the water in its curvature, was (as might have been expected, had I reflected) largely impregnated with the chlorine, and on drawing it up into the mouth, the fluid instantly diffused itself over my fauces, inducing a sensation in the frontal region, similar to that produced by an excess of mustard on our food. I discharged at once the fluid from my mouth, but was instantaneously seized with the most violent attack of sneezing and copious discharge from the nostrils that I had ever experienced, even from the severest cold, and I was obliged to suspend my examination altogether. This state of things continued for more than two hours, when it gradually subsided, but a considerable sense of soreness of the parts remained for several hours. It proved, in short, the most powerful errhine and sternutatory that I remember to have witnessed.

Before I conclude this paper, I will take the occasion to advert to the apparent resemblance of the blood drawn from me in the partially asphyxied state that I have described, both in colour and consistence, with a portion of blood taken from the right side of the heart of a patient, who died of cholera in 1832. This blood, (which I yet have) was received into a greenish-glass phial; but desirous of viewing it to more advantage, I poured it thence into a *white* one. I was greatly struck by its sluggish and tenacious character, as it passed into the latter, trickling down the sides like molasses or copaiba, and leaving a portion adhering to the *green* phial from which it was emptied. So much was this the case, that I was induced to add to what thus remained adherent, as much water as gave it the apparent liquidity of healthy blood, the amount being about four or five parts. The blood in the white phial has (now more than fifteen years corked up and sealed) a rather lighter hue than at first, which may probably arise from the oxygen of the atmospheric air in the upper part of the phial, but not the slightest disposition to coagulation, or a separation of serum, has ever been noticed; it continues to retain its primitive sluggish character. In the other phial to which the water was added, and well shaken, the blood gradually subsided to the bottom in dark grumous flocculi, leaving the water, ultimately, perfectly transparent and untinged.

Whether any practical deductions may be drawn from these facts, I am not aware. Yet, since venesection has been advantageously employed in some cases of intermittent, during the cold stage, and in which a somewhat similar state of the blood has been found to exist, I am inclined to believe that in cholera, in its progress towards its asphyxial or collapsed state, copious venesection would be highly useful, and probably would cut short its continuance and fatal tendency. By copious depletion, less blood would necessarily be carried to the intestines, and thence a less copious amount of its serous portion would be discharged in rice-water stools. Certain it is, that every plan pursued in 1832 appeared inadequate to arrest the discharges, or to check the disease; and the cures, so-called, to me appeared to be more the result of nature than of art! That the whole venous system is engorged, is obvious. No arterial secretion continues, as is evidenced in the almost entire suppression of the urine, whilst the gall-bladder was invariably surcharged with bile, of venous origin. The serous portion of the blood, thus early discharged by stool, renders it impossible for the crassamentum to circulate by the arteries, and from a want of a vis-atergo, it must remain congested in the veins and capillaries, as seen in the livid appearance of the lips, the fingers and other parts of the system. From the same cause (a want of appropriate serous dilution,) the blood ceasing to flow in the lungs, or at least greatly impeded, its arterialization is necessarily imperfect; and should any of it find access to the left side of the heart, it must be more of the venous than of an arterial character, and consequently inadequate to the necessities of the system at large. It may be said that bleeding has often been attempted and failed to obtain any blood! Unquestionably such has been the case—but why? I doubt not, from not being employed at a period sufficiently early, whilst the serum was as yet undiminished. In the preceding case I have stated of myself, it is clear that I was rapidly advancing to that state of asphyxia when bloodletting could not have been accomplished, and I should soon, probably, have been in a state of collapse somewhat like that of cholera. My evacuation was made in less than an hour from the accident that rendered it necessary, and hence, apparently, its successful issue. If any should fear to bleed so largely, let them recollect the amount of blood lost from wounds on the battle field, or from other hæmorrhages, without the loss of life; let them remember that death is at their elbow, watching their movements, and awaiting his prey, if the practitioner is afraid to perform his duty. The system can long maintain its living state with a very small amount of blood, inadequate in a state of health, but fully sufficient in that of syncope or collapse. It is at all events deserving of a fair trial, since it cannot possibly be less successful than that of the thousand nostrums that were tried and found ineffectual amongst us in 1832. Speculation upon speculation has been resorted to, to explain the origin and progress of this extraordinary disease; and not less extensive have been the attempts to fathom the mystery of its nature, essence or proximate cause. Hence the discrepancy of writers as to the operation of its remote causes on the ganglionic nerves, with other idle guesses, the one of equal obscurity with the other. The various recommendations as to its treatment, founded chiefly on such indefinite and unproved doctrines, have consequently, in great measure, proved abortive, and have led to ill-timed delay in a more efficient treatment, from placing too great dependence on deceptive theories. It may be affirmed, I think, that like small pox and many other diseases, we know nothing of their origin, or the modus operandi of their remote causes upon the living system; and that, instead of perplexing ourselves in such occult researches, it would be far preferable to exert ourselves in their treatment, the perfection of which, in respect to many, is, to say the least, extremely problematical.

I am perfectly aware that bleeding has been resorted to in cholera—as may be seen in the very excellent account of the disease, and its treatment, in the Cyclopædia of Practical Medicine, Lond., 1832; or in the 13th vol. of Encycl. Americana, into which it has been transferred. This is a subject of much interest, now that a renewed visit of cholera is beginning to be anticipated; and I may be pardoned for making a few extracts from the work referred to, as worthy of reflection. In speaking of the pulse, after mentioning the symptoms of the attack, it states, that

"The pulse is from the first small, weak and accelerated; and, after a certain interval, but especially on the accession of spasms or severe vomiting, it sinks suddenly, so as to be speedily lost in the external parts. The length of time during which a patient will live in this pulseless state is remarkable. In a case related by Dr. Kellett, the pulse was gone within three hours from the attack; yet the man lived twenty-two hours in that state," &c.

"The state of its (the skin) circulation, and its insensibility, are sometimes strongly denoted by the following circumstances: leeches will not draw blood from it; blisters and other vesicatories will not act; and even the mineral acids and boiling water produce no effect; and some patients are not even sensible of their application," &c.

"The breathing is much affected, being performed either more slowly than usual (sometimes, for instance, in the advanced stage, only at the rate of seven respirations in a minute), or the inspirations are short and sudden," &c.

"The secretions (those of the skin and intestines excepted) are generally suspended."

"The most fatal variety of the disease was denoted by the slightness of the commotion in the system: there was no vomiting; hardly any purging; perhaps only one or two stools, with no perceptible spasm; no pain of any kind; a mortal coldness, with *arrest of the circulation* coming on from the beginning, and the patient dying without a struggle within three or four hours," &c.

"Besides the various and appalling symptoms which indicate general derangement of the action of the solids, there are appearances in the blood, drawn during the *collapsed stage*, showing that the fluids feel the influence of this formidable disease. These appearances are very uniformly expressed by the terms *dark*, *black*, or *tarry*, in regard to colour, and by *thick*, *ropy*, *syrupy*, or *semi-coagulated*, in respect to consistence. This change in the condition of the circulating fluid is fully proved to be in the ratio of the duration of the disease; the blood at the commencement seeming to be nearly or altogether natural, and more or less *rapidly* assuming a morbid state as the malady advances," &c. (variations are noticed here)—the blood in some cases flowed readily from the vein;—"but the reverse was the fact, both with respect to its condition and the manner of its flowing from the arm, in an immense majority of instances.—☞ In general, after a certain quantity of dark, thick blood had been drawn, its *colour became lighter*, *its consistence less thick*, *and the circulation revived*," &c. "Reporters are unanimous in declaring it (the blood) deficient in serum, and destitute of the buffy coat," &c.

"The discharges from patients suffering under this disease were subjected to experiment by Dr. Christie. The secretion consists of two substances, the one a transparent serous fluid, the other an opaque, white coagulum; the former perfectly soluble in cold water, the latter quite insoluble. These matters being submitted to the action of reagents, the fluid part was found to be *pure serum*, and the coagulated portion *fibrin*. The secretion, therefore, as the author remarks, has a composition similar to that of the blood deprived of its colouring matter; but the serum is in *much larger* proportion to the fibrin," &c.

"The urine is suspended throughout the whole course of a choleric stage so intense as we have described,"—(the cold or choleric stage.) The author divides the disease into three stages—"the *incipient*, the *cold* or *choleric*, and the *febrile*."

In the appearances on *dissection*, marks of congestion are found in the *head*, and effusion, &c.—the *lungs* "more frequently *gorged with dark-coloured* blood, so as to resemble liver or spleen"—"*both sides* of the heart in general distended *with dark blood*, and the bronchi are frequently filled with mucus"—"the vessels of the *liver* often much congested, and *pour forth blood copiously* when incisions are made into the organ"—"the *gall-bladder* is turgid with *black bile*, &c.—peritoneum investing the alimentary canal has frequently an inflamed appearance from the *exceedingly loaded* state of its blood-vessels," &c.—"venæ cavæ and *coronary vein* distended with dark-coloured blood"—"the *kidneys* have been observed to partake of the *general congestion of the venous system*, the *bladder*, generally contracted, and either empty, or containing a *small quantity* of urine," &c. "The affection of the alimentary canal is essential and primary, if any part of the disease is so; and it were vain to attempt to trace it to a morbid condition of any other organ or system of organs."

In the cold or choleric stage, the writer points out the circumstances which render bloodletting appropriate or otherwise; "its safe administration *should be early*," &c.; but he points out "three periods of the disease, at which, according to (his) experience, bloodletting may be employed," &c. &c. The whole communication deserves especial consideration from the medical profession at the present period; that, duly estimating its relative merits, &c., they may be prepared to meet this most extraordinary disease, should it again appear amongst us.

Amongst the hitherto inexplicable diseases to which man and many or all of the inferior animals are subject, may be mentioned rabies and that excited by poisonous reptiles—as we have lately seen fatally exemplified in Dr. ——, of New York. Of the particulars of this interesting case, I presume we shall be duly informed. I refer to these—inasmuch as the poison of the rattlesnake and viper have been taken *with impunity* into the stomach, that great centrum sympathiæ! and yet the slightest amount conveyed to the system by the minutest capillary, is capable in a few hours of terminating existence! Both these diseases may be safely styled, opprobria medicorum. In fact, few of the medical profession have an opportunity of seeing either of them;—and when presented to view, have no definite ideas as to their nature, or mode of treatment. One thing alone appears certain: that it is alone through the medium of the blood, that the morbid poison is conveyed internally.

The nature and character of hydrophobia is of extreme interest at the present time to the citizens of this (and perhaps other) cities, from the statements of many of its inhabitants having been bitten by mad dogs. Without entering into a disquisition of a disease, of which we absolutely know nothing, as the conflicting statements of medical writers for ages past in relation to it testify; I shall merely recall to mind, that, in several recorded cases, bloodletting would appear to have been successfully resorted to—although the number is perhaps too small (as yet) on which to found a regular and imperative plan of treatment. Now, in several dissections of persons dying of this disease, the same *dark* appearance of the blood—the same engorged state of the lungs and capillaries—were observed as in cholera; although, unlike it, the blood was *very fluid.* This difference is easily explained, from the one having discharged all its serous portion, whilst it was in full amount in the other: nevertheless, the blackish tint indicates the action of some morbid cause upon it.

The blood is a fluid, sui generis—such as man cannot compound. It is entirely unconnected by adhesion to any part, in its ceaseless flow; and yet is essential to the existence, the nourishment, and vitality of every organized tissue of the body; we know not where, nor how it is created from the inanimate mass of chyle received. It is an imperium in imperio; and is, apparently, governed by laws exclusively its own; for it has no nerves, or actual connexion with them: and hence is devoid of sensibility! It is, in short, the primum vivens, the ultimum moriens of created beings—and in every respect corroborates the scriptural doctrine, that " in blood is the life of man." Hence, and hence only, can we comprehend the high estimate put upon it by our great Creator!—hence, the eating of it was prohibited to His chosen people;—hence, it was the part of the sacrifice that was peculiarly offered to the Divine Majesty;—hence, the *absolute law* laid down by God himself, and *never abrogated*, under the old or new covenant—that "whoever sheds man's blood, by man should his blood be shed;" and to all this may be added—that, in the extensive compass of medical literature, from the earliest period to the present time; amidst the numerous varieties of monsters, recorded by anatomists, of the want of head, brain, STOMACH, lungs, heart, intestines, liver, bladder—in short, of *every individual* organ or tissue—the *blood alone has never*, never been found wanting! This wonderful, and almost self-existing appendage to the microcosm of animal life—this alone, I repeat, has never been found wanting!—and yet our exclusive solidists and ventricular sympathists, cannot admit its appropriate connexion in their physiological and pathological dogmata.

To recur however to hydrophobia, I would earnestly suggest to the practitioners of medicine, to devote some time and serious reflection on this disease; which, like the cholera, may take us unawares;—to abandon the heterogeneous plans of treatment so generally persisted in, and so universally fatal:—to give up all idea of *specifics*, whether mineral, animal, or vegetable;—to abjure even the scutellaria or skullcap;—and, in short, resolve to chalk out some new mode of treatment that may present a plausible and a reasonable hope of success: of such a treatment, I would earnestly present the claims of bloodletting, (to what extent experience must direct,) and accompanied by the operation of bronchotomy, long since proposed by Dr. Physick, in order to sustain respiration, during the violent and prolonged convulsions, with stricture of the glottis, &c., which are concomitants of the disease, and during which the patient usually expires.

